# The efficacy and safety of selective COX-2 inhibitors for postoperative pain management in patients after total knee/hip arthroplasty: a meta-analysis

**DOI:** 10.1186/s13018-020-1569-z

**Published:** 2020-02-05

**Authors:** Mingyang Jiang, Huachu Deng, Xuxu Chen, Yunni Lin, Xiaoyong Xie, Zhandong Bo

**Affiliations:** 1 Department of Bone and Joint Surgery, The First Affiliated Hospital of Guangxi Medical University, Nanning, Guangxi China; 2 Guangxi Medical University, Nanning, Guangxi China

**Keywords:** Selective COX-2 inhibitor, Total knee arthroplasty, Total hip arthroplasty, Meta-analysis

## Abstract

**Background:**

Many selective cyclooxygenase (COX-2) inhibitors are currently used in clinical practice. COX-2 inhibitors have good anti-inflammatory, analgesic, antipyretic effects, and gastrointestinal safety. However, the analgesic effects and adverse reactions of COX-2 after total knee/hip arthroplasty (TKA/THA) are not fully known.

**Objective:**

To evaluate the efficacy and safety of selective COX-2 inhibitors in postoperative pain management in patients receiving TKA/THA.

**Methods:**

Randomized controlled trials (RCTs) were retrieved from medical literature databases. Risk ratios (RR) Std mean difference (SMD) and 95% confidence intervals (CI) were calculated to analyze the primary and safety endpoints.

**Results:**

In total, 18 articles (23 trial comparisons) were retrieved comprising 3104 patients. Among them, 1910 patients (61.5%) were randomized to the experimental group whereas 1194 patients (38.5%) were randomized to the control group. The primary endpoints were the patients’ VAS score at rest or on ambulation (within 3 days). We found that VAS score in patients that received selective COX-2 inhibitor was significantly lower compared to those of the control group.

**Conclusion:**

This meta-analysis shows that selective COX-2 inhibitor therapy is effective, safe, and reliable in relieving postoperative pain of THA/TKA.

## Introduction

Total hip or knee arthroplasty (THA/TKA) is commonly performed to alleviate the symptoms of hip or knee joint dysfunction, promote the recovery of the disease, and improve the quality of life of patients [1]. However, postoperative pain has been a major drawback as it directly affects postoperative rehabilitation of patients [2].

Currently, non-steroidal anti-inflammatory drugs (NSAIDs) are often used for postoperative analgesia [3]. Traditional NSAIDs (such as fotaline, ibuprofen) achieve exert analgesic effects by non-selectively inhibiting cyclooxygenase (COX) [4]. COX comprises two isozyme isomers, COX-1 and COX-2. COX-1 is an inherent housekeeping enzyme [5], mainly distributed in the stomach, kidney, and platelets. It catalyzes the production of physiologically needed prostaglandin E2 (PGE2) that regulates peripheral vascular resistance, platelet aggregation, maintains renal blood flow, and protects gastric mucosa [6]. On the other hand, COX-2 is expressed by monocytes, macrophages, fibroblasts etc.*,* in response to inflammatory stimulation, and thus, it is referred to as inducible enzyme [7]. It is one of the key enzymes that initiate inflammatory reactions and promote inflammatory response leading to tissue injury [8]. NSAIDs, therefore, simultaneously exert anti-inflammation and analgesic effects which also increases the risk of perioperative bleeding and digestive tract symptoms [9]. Selective COX-2 inhibitors not only prevent inflammation and exert analgesic and antipyretic effects, but also protect the gastrointestinal mucosa and are widely used in orthopedic postoperative analgesia [10].

Although COX-2 inhibitors can relieve postoperative pain, their analgesic and adverse effects have not been fully analyzed [11]. This meta-analysis was conducted to explore the efficacy and safety of COX-2 inhibitors in postoperative pain management for patients receiving THA/TKA to provide reference data for clinical guidance.

## Methods

### Search strategy

Two researchers searched for published articles analyzing the efficacy and safety of selective COX-2 inhibitors in postoperative pain management for patients undergoing THA/TKA. We then performed a meta-analysis following the Preferred Reporting Items for Systematic Reviews and Meta-Analyses (PRISMA) guidelines. The randomized controlled trials (RCTs) were systematically searched in databases including PubMed, Embase, the Cochrane Library, Baidu Scholar, Google Scholar, CNKI, and VIP with no restrictions on language or publication date from inception to 12 May 2019. Additional relevant studies were retrieved from reviews, meta-analyses, and other literature. Two authors screened and double-reviewed the retrieved studies. In cases of discrepancies, a third researcher was consulted to obtain a consensus. In this meta-analysis, all data were extracted from previously published studies; thus, patient consent and ethical approval were not required.

### Inclusion and exclusion criteria

We included clinical trials analyzing the efficacy and safety of selective COX-2 inhibitors in patients with THA or TKA and RCTs involving selective COX-2 inhibitors, in which, all patients underwent TKA or THA. The following types of studies were excluded: retrospective trials, animal experiments, non-randomized clinical trials, reviews, series, and case reports; studies with erroneous or incomplete data; studies that did not focus on TKA or THA patients; and studies in which patients were allergic to selective COX-2 inhibitors.

### Endpoints

In this meta-analysis, the primary endpoint was the VAS score within 3 days after surgery. The secondary endpoint was morphine supplementation within 3 days after surgery. The safety endpoints included nausea, vomiting, pruritus, dizziness, fever, edema, lethargy, insomnia, constipation, diarrhea, and headache.

### Data extraction

Two authors independently reviewed the contents of the retrieved studies. The primary endpoints were extracted by two authors and verified by a third author. The data extracted included the following primary information: first author’s name, year of publication, test type/region, sample size, sex ratio, average age, intervention, supplemental analgesic drugs, type of surgery, follow-up time, and endpoints measured in each study. If the content of the studies needed clarification, the first author of the study was contacted. Disagreements were resolved through consensus or by consulting a third author.

### Risk-of-bias assessments

The methodological quality of the included studies was estimated independently by two authors based on the Cochrane Risk of Bias criteria. Seven items were used to assess bias in each trial, i.e., randomization sequence generation, allocation concealment, blinding of participants and personnel, blinding of outcome assessment, incomplete outcome data, selective reporting, and other biases. Each quality item was graded as low risk, high risk, or no clear risk.

### Statistical analysis

Stata (version 12.0, Stata Corp, College Station, Texas) was used to analyze and pool the individual research findings. Pooled results were recorded as risk ratios (RR), Std mean difference (SMD), and 95% confidence intervals (CI) with two-sided *p* values. *p* values < 0.05 were considered to be statistically significant. Heterogeneity was evaluated using the *I*^2^ test. The heterogeneity was considered to be small when *I*^2^ < 50% and substantial when *I*^2^ > 50%. The fixed effect model was used when *I*^2^ < 50%, while the random effect model was used when *I*^2^ > 50%. A funnel plot was generated to examine the publication bias and to explore the sources of heterogeneity if more than ten studies were included to assess this endpoint. Subgroup analysis was performed according to the administration and type of selective COX-2 inhibitor.

## Results

### Characteristics of retrieved studies

**Fig. 1:**
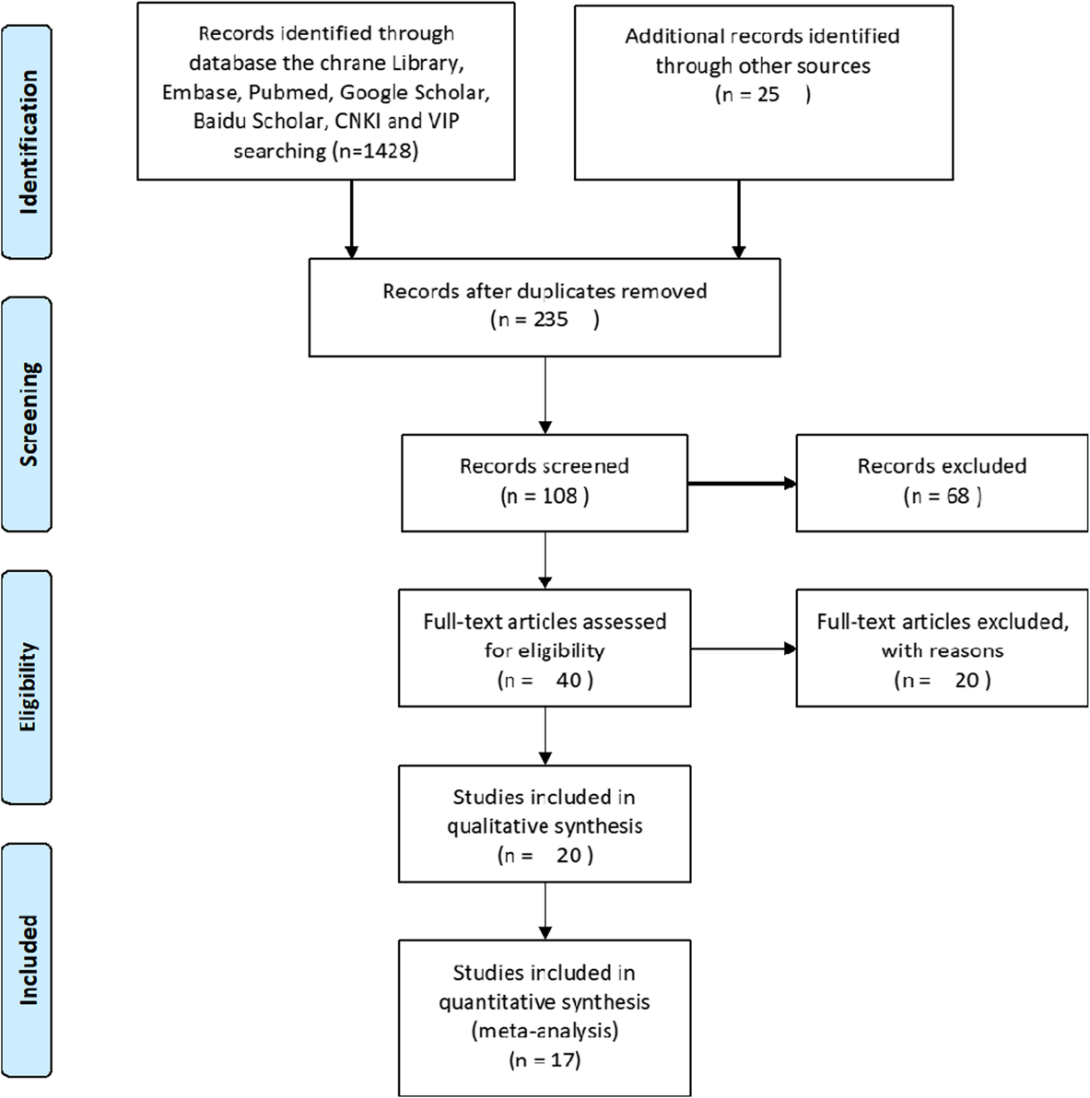
Flow diagram of the study selection process. CNKI, China national knowledge infrastructure; VIP, China Science and Technology Journal Database

In total, 1428 relevant studies were identified in line with the PRISMA guidelines. The titles and abstracts of the studies were screened to exclude irrelevant studies. After reading the full text of the identified studies, we excluded those that did not meet the set criteria. Finally, 17 studies (22 trial comparisons) comprising 2919 patients were enrolled in this meta-analysis as shown in Fig. 1. Among the 2919, 1819 patients (62.3%) were randomized to the selective COX-2 inhibitor group whereas 1100 patients (37.7%) were randomized to the control group. All studies included in this meta-analysis were RCTs. The basic characteristics of the participants in each trial are shown in Table 1.

**Table 1:** Characteristics of studies included in meta-analysis

Author	Year	Country	Sample number		Women (%)		Average age (years)		Intervention		Supplemental analgesic drugs	Operative type	Follow-up	Endpoints
			COX-2 inhibitors	Control	COX-2 inhibitors	Control	COX-2 inhibitors	Control	COX-2 inhibitors	Control				
Buvanendran [12]	2003	USA	35	35	27 (77)	20 (57)	60.0 ± 10.1	62.1 ± 9.8	Rofecoxib: orally, preoperative 50 mg 1 to 2 h and 24 h, postoperative 50 mg/day for 5 days, 25 mg/day for another 8 days	Placebo: orally, for 14 days	Opioid	TKA	14 days	Nausea, vomiting
Rasmussen [13]	2005	USA	80	75	50 (62.5)	43 (57.3)	65.3 ± 10.9	63.9 ± 11.5	Etoricoxib: orally, postoperative 120 mg/day for 7 days	Placebo: orally, for 7 days	Opioid	TKA/THA	7 days	Nausea, dizziness, fever, edema, constipation, diarrhea, headache
Viscusi a [14]	2008	USA	161	159	99 (59.3)	87 (52.1)	60.7 ± 13.5	59.2 ± 13.8	Parecoxib: orally, postoperative 20 mg bid from day 2 to day 5	Placebo: orally, from day 2 to day 5 after surgery	PCA pump or IV bolus: morphine, parecoxib (day 1)	THA	5 days	Morphine consumption, nausea, vomiting, pruritus, dizziness, fever, edema, lethargy, insomnia, constipation, diarrhea, headache
Viscusi b [14]	2008	USA	159	159	90 (54.2)	87 (52.1)	59.3 ± 13.0	59.2 ± 13.8	Parecoxib: orally, postoperative 20 mg/day from day 2 to day 5	Placebo: orally, from day 2 to day 5 after surgery	PCA pump or IV bolus: morphine, parecoxib (day 1)	THA	5 days	Morphine consumption, nausea, vomiting, pruritus, dizziness, fever, edema, lethargy, insomnia, constipation, diarrhea, headache
Ittichaikulthol a [15]	2010	USA	40	40	29 (72.5)	32 (80)	68.33 ± 7.59	64.00 ± 7.41	Celecoxib: 400 mg orally 1 h before surgery	Placebo: orally, 1 h before surgery	Pump: morphine 1 mg	TKA/THA	1 day	Morphine consumption, nausea, vomiting, pruritus
Ittichaikulthol b [15]	2010	USA	40	40	27 (67.5)	32 (80)	68.05 ± 9.75	64.00 ± 7.41	Parecoxib: 40 mg intravenously 1 h before surgery	Placebo: orally, 1 h before surgery	Pump: morphine 1 mg	TKA/THA	1 day	Morphine consumption, nausea, vomiting, pruritus
Li [16]	2010	China	20	20	4 (20)	7 (35)	70 ± 11	68 ± 12	Parecoxib: intravenous injection, postoperative 20 mg/6 h for 2 days	Placebo: intravenous injection, for 2 days	None	TKA	2 days	VAS, nausea, vomiting
Ye [17]	2011	China	30	30	21 (70)	19 (63.33)	60.3 ± 13.4	67.1 ± 11.0	Celecoxib: orally, preoperative 400 mg 8 h before surgery, postoperative 200 mg twice daily for 5 days	No clear	Pump: fentanyl 1 mg, lappaconitine 20 mg, ropivacaine 375 mg for 48 h	TKA/THA	5 days	VAS
Zhou [18]	2011	China	20	20	No clear	No clear	65–74	65–74	Femoral nerve block + parecoxib: 40 mg intravenously 30 min before the end of surgery, 12, 24, and 48 h after surgery	Femoral nerve block	Ropivacaine/8 h for 72 h	TKA	3 days	VAS
Jiang [19]	2012	China	42	41	No clear	No clear	56–78	56–78	Celecoxib: orally, preoperative 200 mg twice daily for 3 days, postoperative 400 mg once and 200 mg twice daily for 7 days	No clear	Pump: sufentanil 0.1 mg for 2 days	TKA	7 days	VAS, vomiting, nausea
Rawal a [20]	2013	USA	222	98	134 (59.8)	56 (57.1)	65.7 ± 8.5	65.2 ± 7.9	Etoricoxib: orally, postoperative 90 mg/day for 7 days	Placebo: orally, postoperative no clear (1 dose), once daily for 7 days	Patient-controlled analgesia (PCA) device: morphine 1 mg	TKA	21 days	Nausea, vomiting, pruritus, dizziness, fever, edema, insomnia, constipation, headache
Rawal b [20]	2013	USA	230	98	139 (60.4)	56 (57.1)	64.7 ± 8.1	65.2 ± 7.9	Etoricoxib: orally, postoperative 120 mg/day for 7 days	Placebo: orally, postoperative no clear(1 dose), once daily, 7 days	Patient-controlled analgesia (PCA) device: morphine 1 mg	TKA	21 days	Nausea, vomiting, pruritus, dizziness, fever, edema, insomnia, constipation, headache
Xu [21]	2015	China	40	40	21 (52.5)	13 (32..5)	55.7 ± 3.1	57.4 ± 1.3	Celecoxib: orally, preoperative 8 h 200 mg, postoperative 200 mg twice daily for 5 days	No clear	Pump: fentanyl 1 mg, lappaconitine 20 mg, ropivacaine 375 mg for 48 h	TKA/THA	5 days	VAS, morphine consumption, vomiting,
Munteanu a [22]	2016	Bucharest	55	55	45 (81.82)	49 (89.09)	66.1 ± 8	64.9 ± 7	Etoricoxib: 120 mg orally at the end of surgery and after 24 h	Placebo: orally, once daily, for 2 days	Morphine	TKA	8 months	Morphine consumption, nausea, vomiting, pruritus
Munteanu b [22]	2016	Bucharest	55	55	44 (80)	49 (89.09)	66.7 ± 7.0	64.9 ± 7	Etoricoxib: 120 mg orally 1 h before surgery and after 24 h	Placebo: orally, once daily, for 2 days	Morphine	TKA	8 months	Morphine consumption, nausea, vomiting, pruritus
Ding a [23]	2016	China	64	40	No clear	No clear	70.3 ± 3.4	69.3 ± 4.1	Celecoxib: orally, postoperative 100 mg twice daily for 6 days	No clear	None	TKA	3 days	VAS, vomiting, nausea
Ding b [23]	2016	China	40	40	No clear	No clear	69.5 ± 5.1	69.3 ± 4.1	Parecoxib: intravenous injection, postoperative 40 mg/12 h for 3 days	No clear	None	TKA	3 days	VAS, vomiting, nausea
Camu [24]	2016	Europe	72	38	42 (58)	19 (50)	65.6 ± 11.0	67.7 ± 9.5	Parecoxib: orally, postoperative 40 mg twice daily for 48 h	Placebo: orally, once daily for 48 h	Pump: morphine 1 mg	THA	2 days	Nausea, vomiting, fever,
Mu [25]	2017	China	310	310	229 (73.9)	227 (73.2)	69.6 ± 6.5	70.5 ± 6.9	Parecoxib: intravenous injection, postoperative 40 mg/12 h for 3 days	Placebo: intravenous injection, postoperative 5 ml/12 h for 3 days	Pump: morphine 0.5 mg/h, 1 mg	TKA/THA	28 months	Morphine consumption, nausea, vomiting
Wang [26]	2017	China	28	27	19 (67.86)	16 (59.26)	66.4 ± 5.7	65.2 ± 5.0	Parecoxib: 40 mg intravenously 30 min before the end of surgery, 6 h, 24 h, and 48 h after surgery	No clear	Pump: fentanyl 8 μg/mL, 1 mg	TKA	3 days	VAS
Yang [27]	2017	China	30	30	24 (80)	25 (83.33)	65.53 ± 6.28	66.55 ± 5.50	Parecoxib: postoperative 40 mg/12 h for 3 days, intravenous injection, celecoxib: orally, 200 mg/12 h until 6 weeks after surgery	Placebo: intravenous injection/orally, 6 weeks	Pump: morphine, 60 mg	TKA	6 weeks	VAS, nausea, vomiting, pruritus, headache
Bian [28]	2018	China	46	42	34 (73.91)	30 (75.43)	66.64 ± 7.27	66.12 ± 8.34	Parecoxib: orally, preoperative 40 mg, postoperative 40 mg/12 h	Placebo: orally, preoperative 2 ml, postoperative 2 ml/12 h	Pump: morphine	TKA	14 days	Nausea, vomiting, dizziness, lethargy, headache

*VAS* visual analog scale, *THA* total hip arthroplasty, *TKA* total knee arthroplasty

### Evaluating literature quality

The Cochrane Risk of Bias criteria was used to evaluate the quality of the enrolled studies, which was conducted by two researchers. All included studies were randomized controlled trials. In summary, 17 studies [12–28] described random sequence generation and allocation concealment. Nine studies [12–14, 20, 22, 24, 25, 27, 28] described the blinding of participants and personnel. Nine studies [12–14, 20, 22, 24, 25, 27, 28] described the blinding of outcome assessment. None of the studies described other biases. The literature quality scores of each study are shown in Table 2. The random effect model was applied to determine the level of heterogeneity among the articles.

**Table 2:** Assessment of methodological quality of included studies

Study	Random allocation	Hidden distribution	Blind method	Incomplete outcome data	Selective reporting of results	Other bias	Quality grade
Buvanendran [12]	Randomized	No clear	Double-blind	Low	Low	Low	C
Rasmussen [13]	Randomized	No clear	Double-blind	Low	Low	Low	B
Viscusi a [14]	Randomized	No clear	Double-blind	Low	Low	Low	B
Viscusi b [14]	Randomized	No clear	Double-blind	Low	Low	Low	B
Ittichaikulthol a [15]	Randomized	No clear	No clear	Low	Low	Low	C
Ittichaikulthol b [15]	Randomized	No clear	No clear	Low	Low	Low	C
Li [16]	Randomized	No clear	No clear	Low	Low	Low	C
Ye [17]	Randomized	No clear	No clear	Low	Low	Low	C
Zhou [18]	Randomized	No clear	No clear	Low	Low	Low	C
Jiang [19]	Randomized	No clear	No clear	Low	Low	Low	C
Rawal a [20]	Randomized	No clear	Double-blind	Low	Low	Low	B
Rawal b [20]	Randomized	No clear	Double-blind	Low	Low	Low	B
Xu [21]	Randomized	No clear	No clear	Low	Low	Low	C
Munteanu a [22]	Randomized	No clear	Double-blind	Low	Low	Low	A
Munteanu b [22]	Randomized	No clear	Double-blind	Low	Low	Low	A
Ding a [23]	Randomized	No clear	No clear	Low	Low	Low	C
Ding b [23]	Randomized	No clear	No clear	Low	Low	Low	C
Camu [24]	Randomized	No clear	Double-blind	Low	Low	Low	A
Mu [25]	Randomized	No clear	Double-blind	Low	Low	Low	A
Wang [26]	Randomized	No clear	No clear	Low	Low	Low	C
Yang [27]	Randomized	No clear	Double-blind	Low	Low	Low	C
Bian [28]	Randomized	No clear	Double-blind	Low	Low	Low	B

### Primary endpoints

#### VAS score at rest within 3 days post-surgery

**Fig. 2:**
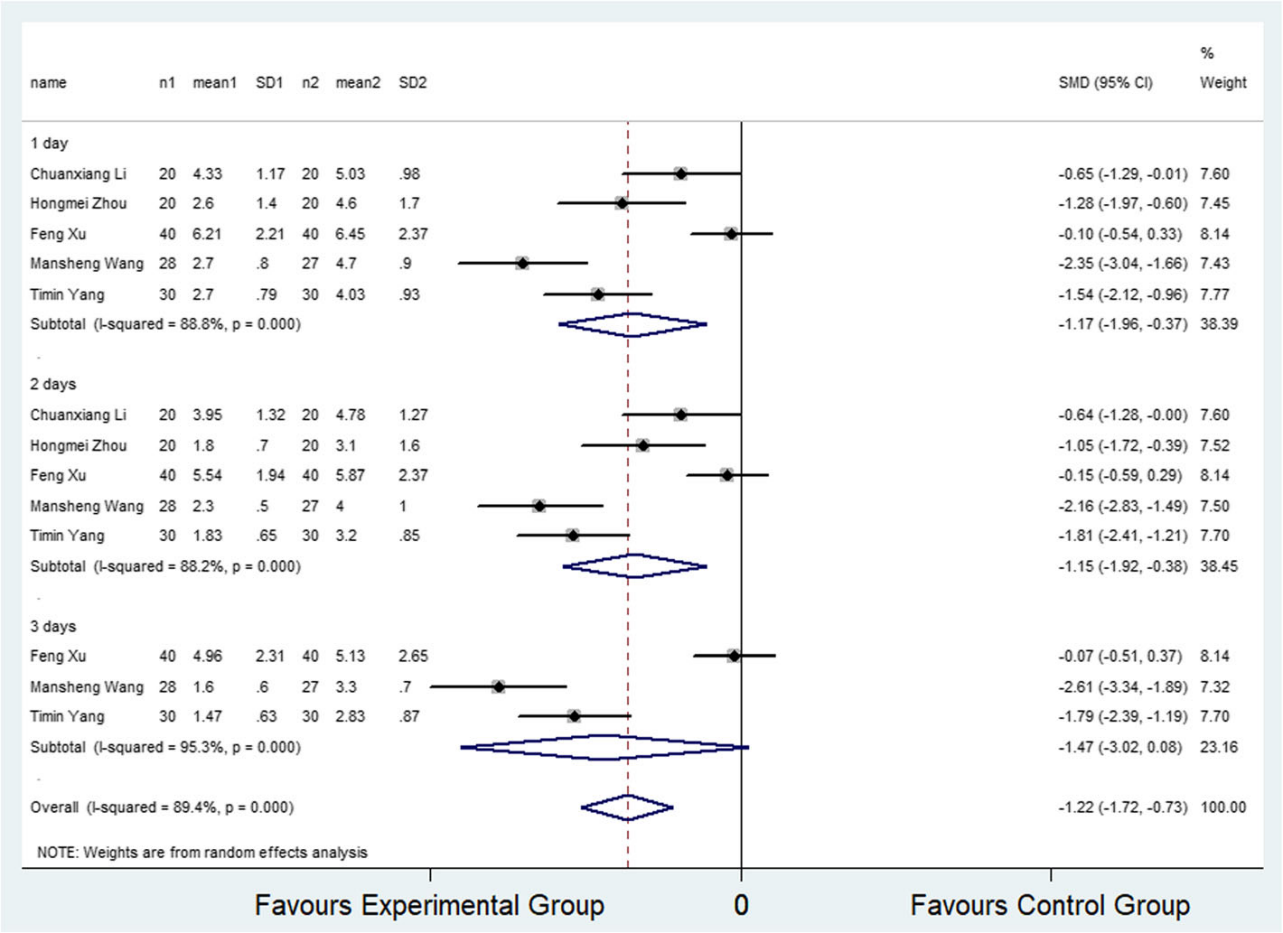
Comparison of VAS score at rest within 3 days after surgery between the selective COX-2 inhibitor group and the control group. SMD, standardized mean difference

Five studies [16, 18, 21, 26, 27] (5 trial comparisons) reported VAS score at rest within 3 days after surgery. In total, VAS score at rest was provided for 275 patients, of whom 138 were assigned to the selective COX-2 inhibitor group and 137 were assigned to the control group. The result showed that the VAS scores at rest in the selective COX-2 inhibitor group were significantly lower compared to the control group (SMD − 1.22, 95% CI − 1.72 to − 0.73, *I*^2^ = 89.4%) as shown in Fig. 2. The random effect model was applied to determine the level of heterogeneity among the articles. The subgroup analysis revealed that the VAS scores at rest in selective COX-2 inhibitor group were significantly lower than that in the control group at 24 and 48 h (SMD − 1.17, 95% CI − 1.96 to − 0.37; SMD − 1.15, 95% CI − 1.92 to − 0.38). However, the VAS scores were not significantly different between the selective COX-2 inhibitor and control groups at 72 h (SMD − 1.47, 95% CI − 3.02 to − 0.08).

#### VAS scores after ambulation within 3 days post-surgery

**Fig. 3:**
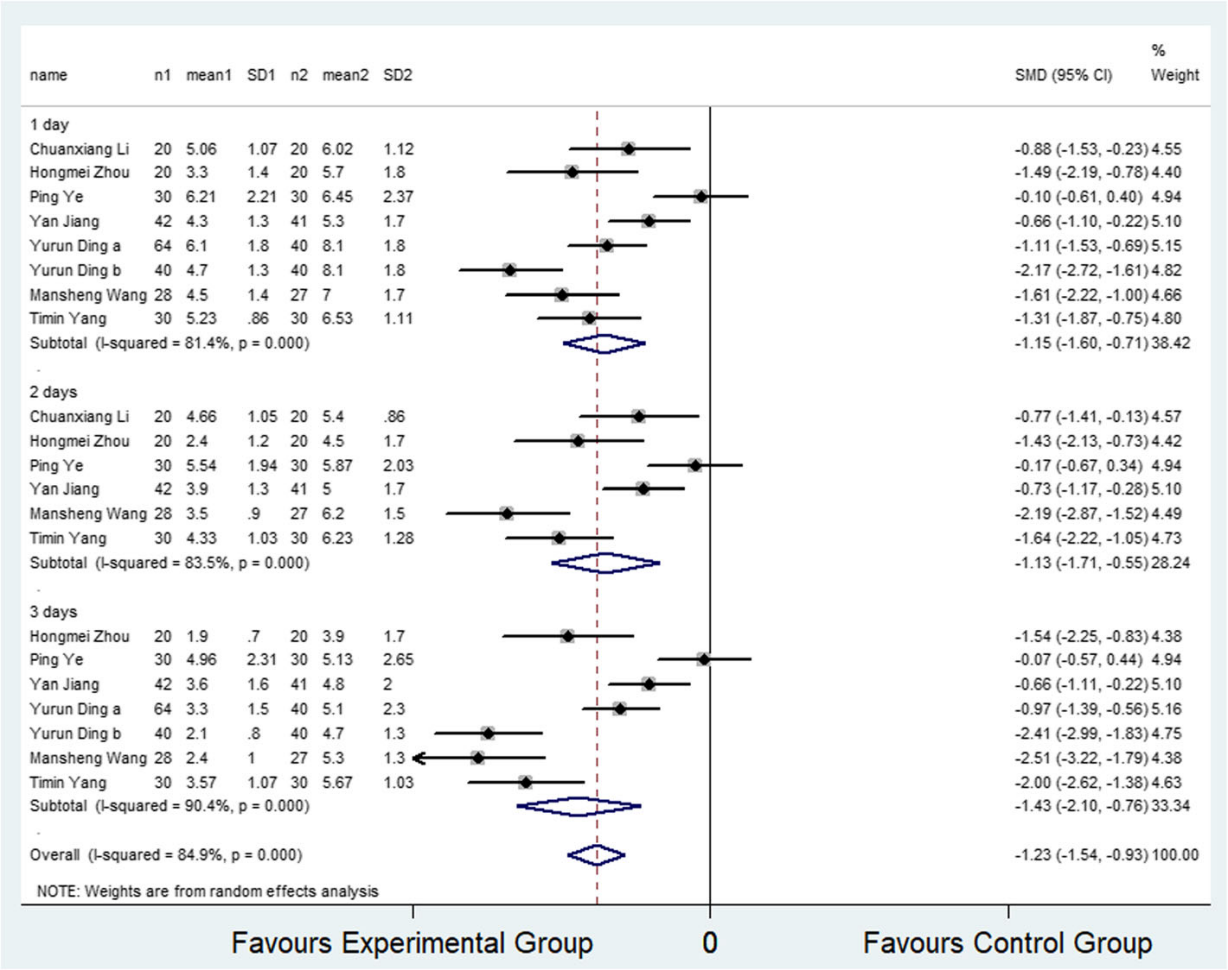
Comparison of VAS score on ambulation within 3 days after surgery between the selective COX-2 inhibitor group and the control group. SMD, standardized mean difference

Seven studies [16–19, 23, 26, 27] (8 trial comparisons) reported VAS scores for ambulation within 3 days after surgery. In total, VAS scores after ambulation were reported for 482 patients, of which 274 were assigned to the selective COX-2 inhibitor group and 208 were assigned to the control group. It was observed that VAS scores after ambulation in the selective COX-2 inhibitor group were significantly lower than that of the control group (SMD − 1.23, 95% CI − 1.54 to − 0.93, *I*^2^ = 84.9%) as shown in Fig. 3. The random effect model was applied to determine the level of heterogeneity among the articles. The subgroup analysis revealed that VAS scores after ambulation in selective COX-2 inhibitor group were significantly lower than that of the control group at 24, 48, and 72 h (SMD − 1.15, 95% CI − 1.60 to − 0.71; SMD − 1.13, 95% CI − 1.71 to − 0.55; SMD − 1.43, 95% CI − 2.10 to − 0.76).

In the administration subgroup analysis, it was found that the VAS scores in selective COX-2 inhibitor group were significantly lower than that of the control group when selective COX-2 inhibitors were injected (SMD − 1.55, 95% CI − 2.09 to − 1.01; SMD − 1.46, 95%CI − 2.28 to − 0.64; SMD − 2.17, 95%CI − 2.74 to − 1.59) within 24 and 72 h. Similar results were obtained when selective COX-2 inhibitors were orally administered (SMD − 0.64, 95%CI − 1.20 to − 0.08; SMD − 0.59, 95%CI − 1.09 to − 0.08). There was no significant difference when selective COX-2 inhibitors were orally administered (SMD − 0.46, 95% CI − 1.01 to 0.09).

In the medication type subgroup analysis, it was observed that the VAS scores in the selective COX-2 inhibitor group were significantly lower than that of the control group when parecoxib (SMD − 1.55, 95% CI − 2.09 to − 1.01; SMD − 1.46, 95%CI − 2.28 to − 0.64; SMD − 2.17, 95%CI − 2.74 to − 1.59) was administered within 24 and 72 h. Similar results were obtained when celecoxib was administered (SMD − 0.64, 95%CI − 1.20 to − 0.08; SMD − 0.59, 95%CI − 1.09 to − 0.08). There was no significant difference when using celecoxib (SMD − 0.46, 95% CI − 1.01 to 0.09).

### Secondary endpoints

#### Morphine supplementation within 3 days after surgery

**Fig. 4:**
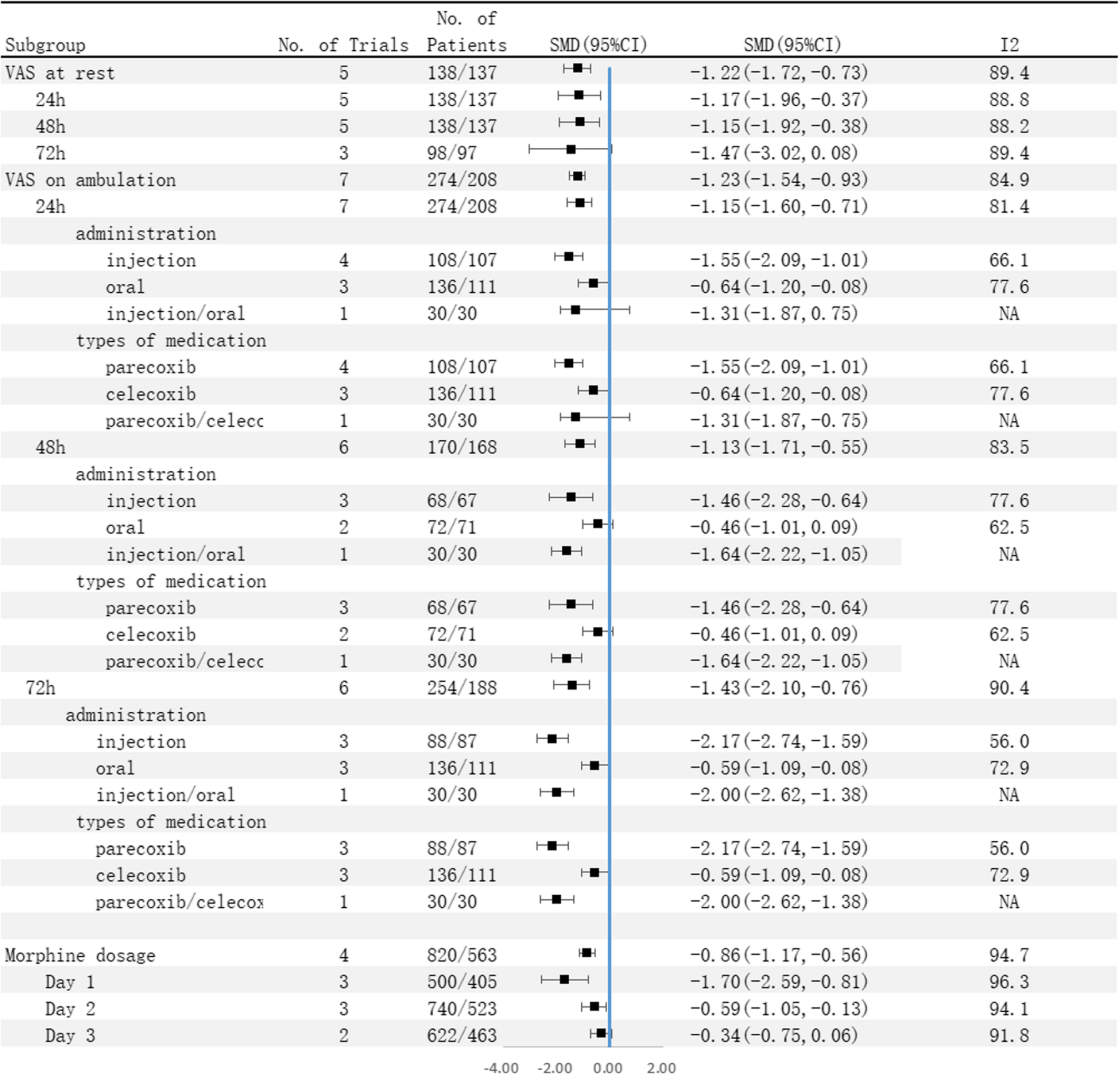
Comparison of VAS score on ambulation and morphine supplement within 3 days after surgery between the selective COX-2 inhibitor group and the control group (subgroup analysis). SMD, standardized mean difference

Four studies [14, 15, 22, 25] (7 trial comparisons) reported morphine supplementation within 3 days after surgery. In total, 1383 patients received morphine supplementation, among whom 820 were assigned to the selective COX-2 inhibitor group and 563 were assigned to the control group. Figure 4 shows that the quantity of morphine supplementation in the selective COX-2 inhibitor group was significantly lower than that of the control group (SMD − 0.86, 95% CI − 1.17 to − 0.56, *I*^2^ = 94.7%). The random effect model was applied to determine the level of heterogeneity among the articles. In subgroup analysis, the quantity of morphine supplementation in the selective COX-2 inhibitor group was significantly lower than that of the control group at 24 and 48 h (SMD − 1.70, 95% CI − 2.59 to − 0.81, *I*^2^ = 96.3%; SMD − 0.59, 95% CI − 1.05 to − 0.13, *I*^2^ = 94.1%). Notably, the difference between the two groups in morphine supplementation at 72 h was not significant (SMD − 0.34, 95% CI − 0.75 to 0.06, *I*^2^ = 91.8%).

### Safety endpoints

Further analysis revealed that administration of selective COX-2 inhibitors significantly reduced the incidence of nausea (14.3% vs 17.5%) (RR 0.72, 95% CI 0.61 to 0.85), vomiting (8.6% vs 12.8%) (RR 0.68, 95% CI 0.54 to 0.85), and fever (7.3% vs 17.6%) (RR 0.36, 95% CI 0.27 to 0.47).

Moreover, there was no significant difference between the selective COX-2 inhibitor group and the control group in the occurrence of pruritus (4.5% vs 4.7%) (RR 0.85, 95% CI 0.53 to 1.36), dizziness (5.3% vs 5.8%) (RR 0.90, 95% CI 0.59 to 1.37), edema (2.8% vs 3.9%) (RR 0.92, 95% CI 0.53 to 1.59), lethargy (3.3% vs 5.5%) (RR 0.57, 95% CI 0.29 to 1.14), insomnia (7.4% vs 9.3%) (RR 0.70, 95% CI 0.47 to 1.02), constipation (12.8% vs 14.2%) (RR 0.93, 95% CI 0.71 to 1.22), diarrhea (1.8% vs 3.0%) (RR 0.63, 95% CI 0.25 to 1.57), and headache (4.1% vs 5.9%) (RR 0.79, 95% CI 0.50 to 1.25).

### Publication bias and sensitivity analysis

A funnel plot showed that there was bias among the enrolled studies as shown in Additional file 1: Figure S1–S3. The results of the sensitivity analysis are shown in Additional file 2: Figure S4–S11.

## Discussion

Postoperative pain severely limits limb movement and functional exercise after THA/TKA, and this compromises the recovery of postoperative joint function [29]. Currently, many selective COX-2 inhibitors are used in clinical practice [30]. These drugs have good anti-inflammatory, analgesic, antipyretic effects without causing gastrointestinal mucosal damage [31]. So far, the analgesic effects and adverse reactions of selective COX-2 inhibitors after THA/TKA are not fully understood.

In recent years, research on of the application of selective COX-2 inhibitors has received tremendous attention. A review conducted by Parvizi et al. [32] provided strong evidence in favor of using NSAIDs and selective COX-2 inhibitors as part of the multimodal treatment plan in pain management. A meta-analysis by Ji et al. [33] indicated that preoperative administration of COX-2 inhibitor effectively improves postoperative analgesia, reduces the consumption of morphine, and lessens the incidence of pruritus. Other meta-analyses [34, 35] comparing the efficacy and safety of selective COX-2 inhibitors versus non-selective COX-2 inhibitors in the prevention of HO after THA revealed that selective COX-2 inhibitors are as effective as non-selective COX-2 inhibitor in preventing Heme oxygenase (HO) after THA. Of note, selective COX-2 inhibitors were associated with fewer gastrointestinal side effects than non-selective COX-2 inhibitor. A preliminary study by Oh et al. [36], the efficacy of a selective COX-2 inhibitor in early postoperative pain control, pain management satisfaction level, and incidence of systemic adverse effects in patients undergoing arthroscopic rotator cuff repair were evaluated. This study concluded that, although the selective COX-2 inhibitors have similar postoperative analgesic effects as other non-steroidal anti-inflammatory drugs and opioids, they might negatively affect tendon-to-bone healing after surgical repair, and thus, they should not be used for postoperative analgesia. Vastel et al. [37] compared the efficacy of celecoxib versus ketoprofen. They reported that celecoxib and ketoprofen had equivalent efficacy in reducing peri-prosthetic ossifications. Gong et al. [38] conducted an RCT to evaluate the efficacy of muscle relaxants and celecoxib in early recovery after total knee arthroplasty (TKA). They found that co-application of celecoxib and eperisone with a low dose of morphine provided good and safe pain control in patients undergoing TKA. They summarized the efficacy of selective COX-2 inhibitor in early postoperative pain control, satisfaction with pain management, and incidence of systemic adverse effects in patients undergoing arthroscopic rotator cuff repair. Despite these findings, whether selective COX-2 inhibitors are effective for clinical postoperative pain management after TKA/THA deserves further analysis.

Unlike previous studies which separately explored TKA or THA, our meta-analysis examined both conditions simultaneously. This is also the first meta-analysis to explore the efficacy and safety of selective COX-2 inhibitors for postoperative pain management after TKA/THA. The primary endpoints show that the VAS scores at rest or on ambulation (within 3 days) in patients receiving selective COX-2 inhibitor are significantly lower than that of control subjects (SMD − 1.22, 95% CI − 1.72 to − 0.73; SMD − 0.86, 95% CI − 1.17 to − 0.56). Analysis of secondary endpoints shows that patients receiving selective COX-2 inhibitor require a smaller amount of morphine supplementation within 3 days after surgery (SMD − 0.86, 95% CI − 1.17 to − 0.56) compared to the control subjects. Analysis of safety endpoints show that patients receiving selective COX-2 inhibitor have lower incidence of nausea, vomiting, and fever (RR 0.72, 95% CI 0.61 to 0.85; RR 0.68, 95% CI 0.54 to 0.85; RR 0.36, 95% CI 0.27 to 0.47) compared to the control subjects. Notably, the occurrence of pruritus, dizziness, lethargy, edema, insomnia, constipation, diarrhea, and headache were not significantly different between patients in the selective COX-2 inhibitor group and control group.

Results of the primary endpoints were highly heterogeneous, and thus, we performed sensitivity analyses to identify the sources of heterogeneity. The results show that the heterogeneity did not come from a certain article. The subgroup analyses performed according to the type of selective COX-2 inhibitor and mode of administration did not identify the source of heterogeneity. We thus postulate that heterogeneity among the studies may be caused by the small sample size in the studies.

The potential clinical implications of this meta-analysis are as follows: (1) This is the first study focusing on the efficacy and safety of selective COX-2 inhibitors for postoperative pain management after TKA/THA. A total of 17 RCTs were enrolled covering 2919 participants, which is a larger sample size compared to previous studies. (3) Sensitivity analyses and subgroup analyses were conducted to identify sources of heterogeneity and explore the influence of sample size on the overall effect. (4) We evaluated 13 indicators, including VAS score, morphine supplementation within 3 days after surgery, and the incidence of nausea, vomiting, fever, pruritus, dizziness, lethargy, edema, insomnia, constipation, diarrhea, and headache, making this meta-analysis more comprehensive than previous studies.

**Fig. 5:**
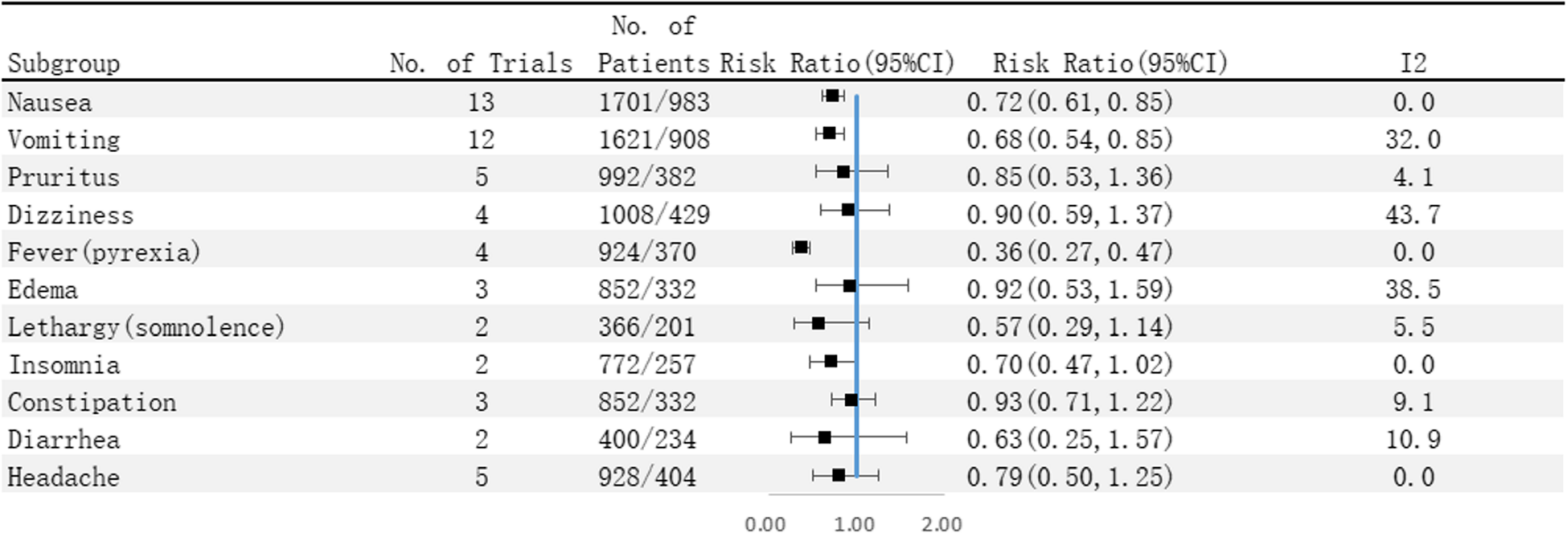
Incidence of adverse reaction between the selective COX-2 inhibitor group and the control group. SMD, standardized mean difference

The limitations of this study are as follows: (1) Several baseline characteristics (diabetes, hypertension, older age, or other drug use) were not considered, and this may introduce mixed bias. (2) We used the outcome events reported in the identified studies. Therefore, it is difficult to assess the effect of these baseline characteristics on the results of this meta-analysis. (3) We did not explore the interactions among the subgroups because of the limitations inherent in the included studies. (4) The primary endpoints results were highly heterogeneous, and sensitivity analyses and subgroup analyses failed to identify the source of heterogeneity. (5) Because of the limited number of articles, the operative type and follow-up period subgroups were meaningless. (6) Only 9 studies described the blinding method used, pointing to the existence of mixed bias. (7) Many studies identified were small (with less than 1000 patients on average), and most of them were published in China and the USA. Thus, high-quality RCTs with large sample sizes are required to determine whether our results are applicable to other geographical regions. (8) Since most of the studies included provided interventions and supplementary analgesic drugs in the control group, the handling of labor pains is actually multimodal (although the authors of the included articles have controlled variables as much as possible). This may lead to high heterogeneity and ultimately affect the results. (9) The primary endpoints were analyzed at 24, 48, and 72 h after operation. However, given that the included studies did not mention the time point at which adverse reactions were recorded, subgroup analysis of secondary endpoints was not conducted according to time (Fig. 5).

This meta-analysis reveals that selective COX-2 inhibitors significantly reduce VAS scores at rest or on ambulation, and decreases morphine consumption within 3 days after surgery, the incidence of nausea, vomiting, and fever. This indicates that selective COX-2 inhibitors are highly effective and safe for postoperative pain control after TKA/THA.

## Supplementary information

**Additional file 1: Figure S1.** Comparison of nausea between the selective COX-2 inhibitor group and the control group. (funnel plot). RR= Risk Ratio. **Figure S2.** Comparison of vomiting between the selective COX-2 inhibitor group and the control group. (funnel plot). RR= Risk Ratio. **Figure S3.** Comparison of pruritus between the selective COX-2 inhibitor group and the control group. (funnel plot). RR= Risk Ratio.

**Additional file 2: Figure S4.** Comparison of VAS score at rest within 3 days after surgery between the selective COX-2 inhibitor group and the control group. (sensitivity analysis). SMD= standardized mean difference. **Figure S5.** Comparison of VAS score at rest at 24 hours after surgery between the selective COX-2 inhibitor group and the control group. (sensitivity analysis). SMD= standardized mean difference. **Figure S6.** Comparison of VAS score at rest at 48 hours after surgery between the selective COX-2 inhibitor group and the control group. (sensitivity analysis). SMD= standardized mean difference. **Figure S7.** Comparison of VAS score at rest at 72 hours after surgery between the selective COX-2 inhibitor group and the control group. (sensitivity analysis). SMD= standardized mean difference. **Figure S8.** Comparison of VAS score on ambulation within 3 days after surgery between the selective COX-2 inhibitor group and the control group. (sensitivity analysis). SMD= standardized mean difference. **Figure S9.** Comparison of VAS score on ambulation at 24 hours after surgery between the selective COX-2 inhibitor group and the control group. (sensitivity analysis). SMD= standardized mean difference. **Figure S10**. Comparison of VAS score on ambulation at 48 hours after surgery between the selective COX-2 inhibitor group and the control group. (sensitivity analysis). SMD= standardized mean difference. **Figure S11.** Comparison of VAS score on ambulation at 72 hours after surgery between the selective COX-2 inhibitor group and the control group. (sensitivity analysis). SMD= standardized mean difference.

## Data Availability

Not applicable

## Abbreviations

CI: Confidence intervals
COX: Cyclooxygenase
NSAIDs: Non-steroidal anti-inflammatory drugs
PRISMA: Reporting Items for Systematic Reviews and Meta-Analyses
RCTs: Randomized controlled trials
RR: Risk ratios
SMD: Std mean difference
TKA/THA: Total knee/hip arthroplasty
VAS: Visual analog scale
