# Supplementary figures and images for: The efficacy and safety of selective COX-2 inhibitors for postoperative pain management in patients after total knee/hip arthroplasty: a meta-analysis
Source: J Orthop Surg Res. 2020 Feb 5;15:39. doi: 10.1186/s13018-020-1569-z (PMC7003344; doi:10.1186/s13018-020-1569-z)

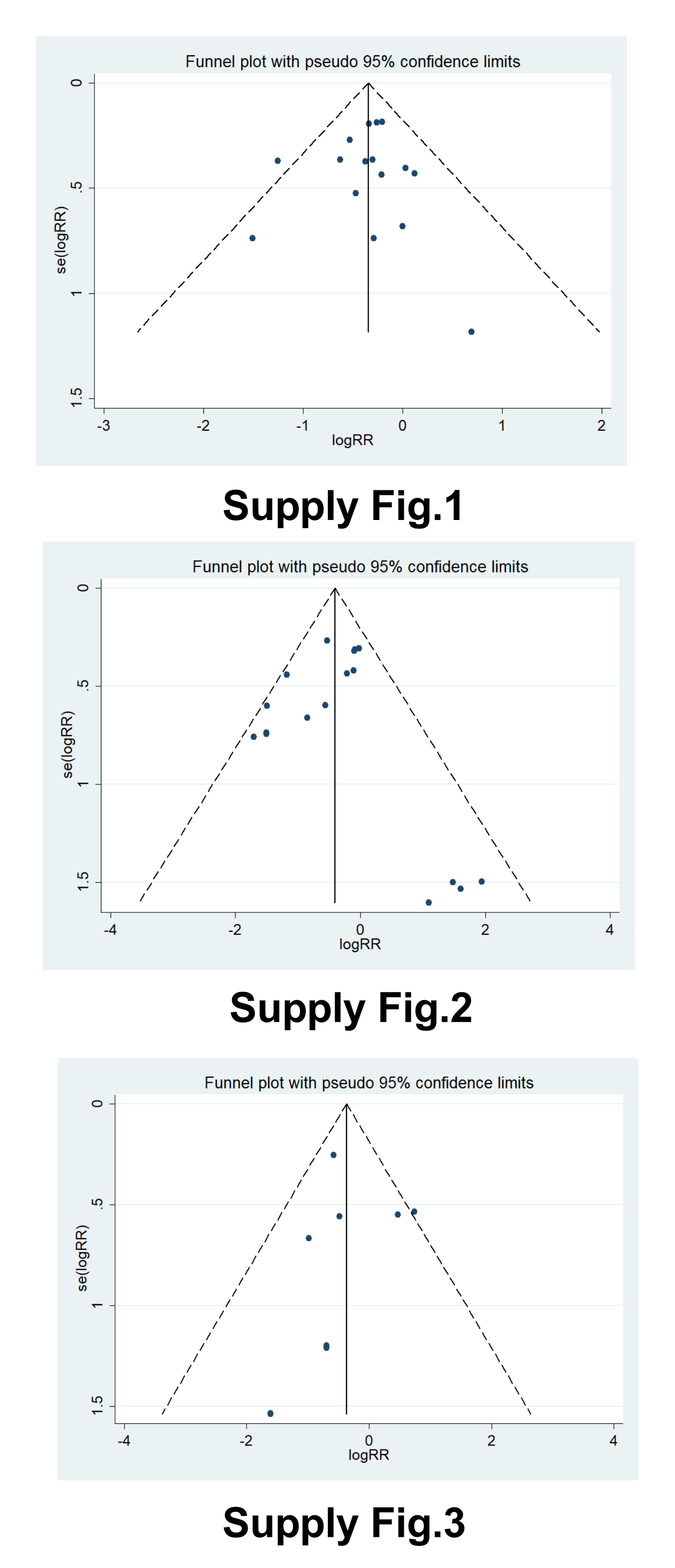

Supplement: Supplementary file 1 — Additional file 1: Figure S1. Comparison of nausea between the selective COX-2 inhibitor group and the control group. (funnel plot). RR= Risk Ratio. Figure S2. Comparison of vomiting between the selective COX-2 inhibitor group and the control group. (funnel plot). RR= Risk Ratio. Figure S3. Comparison of pruritus between the selective COX-2 inhibitor group and the control group. (funnel plot). RR= Risk Ratio. [file 13018_2020_1569_MOESM1_ESM.tif]

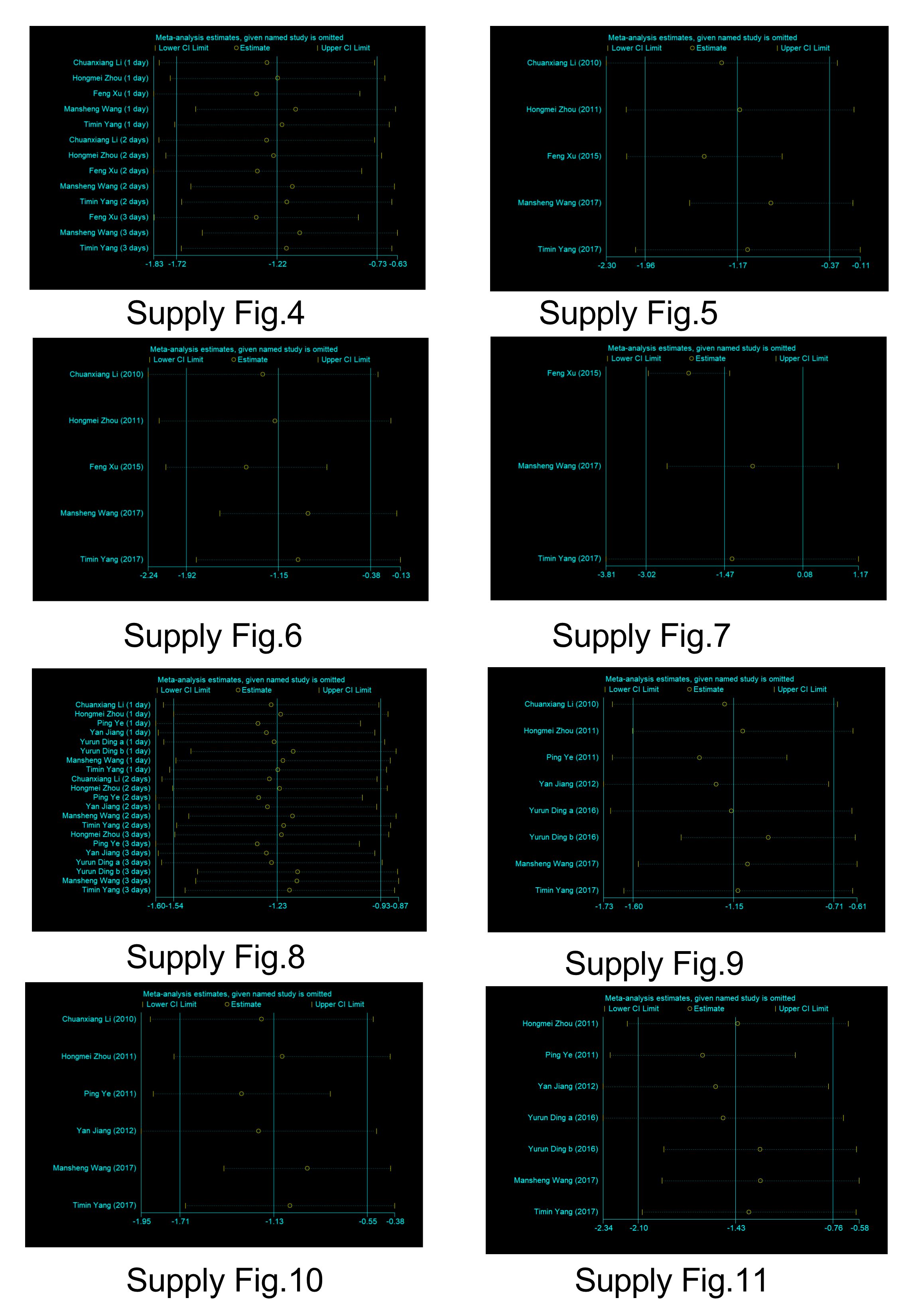

Supplement: Supplementary file 2 — Additional file 2: Figure S4. Comparison of VAS score at rest within 3 days after surgery between the selective COX-2 inhibitor group and the control group. (sensitivity analysis). SMD= standardized mean difference. Figure S5. Comparison of VAS score at rest at 24 hours after surgery between the selective COX-2 inhibitor group and the control group. (sensitivity analysis). SMD= standardized mean difference. Figure S6. Comparison of VAS score at rest at 48 hours after surgery between the selective COX-2 inhibitor group and the control group. (sensitivity analysis). SMD= standardized mean difference. Figure S7. Comparison of VAS score at rest at 72 hours after surgery between the selective COX-2 inhibitor group and the control group. (sensitivity analysis). SMD= standardized mean difference. Figure S8. Comparison of VAS score on ambulation within 3 days after surgery between the selective COX-2 inhibitor group and the control group. (sensitivity analysis). SMD= standardized mean difference. Figure S9. Comparison of VAS score on ambulation at 24 hours after surgery between the selective COX-2 inhibitor group and the control group. (sensitivity analysis). SMD= standardized mean difference. Figure S10. Comparison of VAS score on ambulation at 48 hours after surgery between the selective COX-2 inhibitor group and the control group. (sensitivity analysis). SMD= standardized mean difference. Figure S11. Comparison of VAS score on ambulation at 72 hours after surgery between the selective COX-2 inhibitor group and the control group. (sensitivity analysis). SMD= standardized mean difference. [file 13018_2020_1569_MOESM2_ESM.tif]
